# Indwelling Pleural Catheters for Malignant Pleural Effusion: A Time for Action

**DOI:** 10.1097/NHH.0000000000001023

**Published:** 2021-11-05

**Authors:** Mary C. Vrtis, Eileen DeCesare, Rebecca S. Day

**Affiliations:** **Mary C. Vrtis, PhD, MSN, RN, OCN, NEA-BC, CDP, CADDCT**, is a Website owner, nurses-hands.org, and Vice President of Clinical Operations and Regulatory Compliance, Professional Healthcare Resources, Annandale, Virginia.; **Eileen DeCesare, MS, RN, CNAA, LNC, NE-BC**, is President, CEO Emeritus, Professional Healthcare Resources, Annandale, Virginia.; **Rebecca S. Day, MSN, Ma Ed, RN, CCM**, is Care Coordinator Oncology and Hospice, Federal Employee Program, Care First Blue Cross Blue Shield, Washington, D.C.

## Abstract

Malignant pleural effusion (MPE) resulting from metastatic spread to the pleura frequently occurs in patients with primary lung, breast, hematological, gastrointestinal, and gynecological cancers. These effusions tend to reaccumulate quickly, and the patient requires increasingly frequent thoracentesis. An indwelling pleural catheter allows for dramatic improvement in quality of life as the patient has the power to ease her/his own suffering by draining the effusion at home when shortness of breath and/or chest pain intensifies. Patients with MPE need home healthcare support to address symptom management related to complications of advanced metastatic cancer and antineoplasm treatment regimens. The financial obstacles for the home healthcare agency are explored by using agency supply costs, per visit costs, and the patient-driven groupings reimbursement mode grouper to estimate reimbursement. Care for a home healthcare patient with MPE costs Medicare approximately $64.50 per day, markedly less than costs for hospitalization and outpatient thoracentesis. Unfortunately, agencies must absorb the cost of vacuum drainage bottles. Whereas a small positive balance of $291 was estimated for the first 30-day posthospital episode, losses were estimated at $1,185 to $1,633 for subsequent 30-day episodes. Absorbing these costs has become extremely difficult as home healthcare agencies are experiencing unprecedented COVID-19 infection control and staffing-related costs.

Mrs. M, a 66-year-old woman with stage IV breast cancer, was referred to home healthcare following a 5-day acute care hospitalization for severe dyspnea. She had been relatively stable until 3 weeks ago when she experienced worsening midthoracic back pain and shortness of breath. Follow-up diagnostics showed three metastatic lung masses and bone metastasis to the thoracic spine. During her oncology office visit, Mrs. M demonstrated severe shortness of breath while walking and required rest periods every 10 to 15 feet, her oxygen saturation dropped to 83%, oxygen was administered, and she was transported to the hospital. A 1,500 mL pleural effusion was drained by thoracentesis with a marked decrease in dyspnea. Malignant cells were present in the exudate. An indwelling pleural catheter ([IPC] Figure 1) was placed during the hospitalization and she and her family were instructed on how to perform the vacuum-assisted drainage at the hospital.

**Figure 1.**
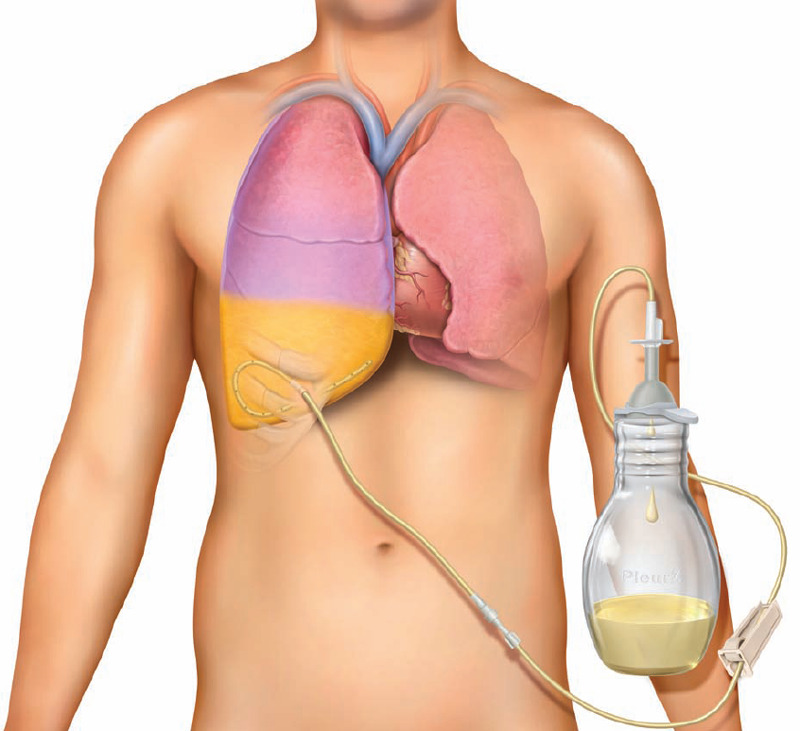
PleurXTM indwelling pleural catheter Courtesy and © Becton, Dickinson and Company. Reprinted with permission.

Mrs. M has advanced disease and has exhausted most of her treatment options (surgery, adjuvant chemo, and two targeted therapies) and any further chemo or targeted therapy would be palliative. The focus of care is aggressive symptom management to improve quality of her life. She is admitted to a home health palliative care program as she wants to continue treatment and is not interested in hospice.

## Malignant Pleural Effusion

Under normal circumstances, the space between the visceral pleura that covers the lung and the parietal pleura that is attached to the chest wall contains about 0.3 mL/kg of fluid (approximately 20 mL). In the absence of pathology, the amount of fluid produced by the pleura and the amount that is reabsorbed stays in balance. A pleural effusion develops when production exceeds reabsorption. Heart failure, pneumonia, and cancer are the most common causes of pleural effusions (Mercer et al., 2019).

Malignant pleural effusion (MPE) results from metastatic spread of cancer, most often in patients with lung, breast, hematological, gastrointestinal, and gynecological tumors. In 2012, 126,825 patients with a primary or secondary diagnosis of MPE were admitted to U.S. hospitals. In a study by Taghizadeh et al. (2017) with a median hospital charge of $42,376 and a 5.5-day median length of stay, the total bill for care was over $5 billion dollars. Chest tubes are placed to drain the effusions in 31.9% of patients during hospitalization (Fortin et al., 2018); however, we are not able to determine how many were IPCs established for home drainage. A second study based on 2014 data from 108,824 patients with MPE admitted to hospitals reported a 25.6% readmission rate with a total cost of over $400 million (Mitchell et al., 2020). Home health and hospice care can be provided at markedly reduced costs. There are significant financial challenges related to home healthcare for patients with IPCs which will be discussed later in this article.

With MPE, cancer cells break from the primary tumor and metastasize to the pleura via the circulatory system or through direct tumor invasion from adjacent tissues such as the breast or lung. The diseased pleural cells produce large amounts of proteinaceous fluid that accumulates between the visceral and parietal pleura. The high protein content is likely due to leakage of plasma proteins and the oncotic pressure changes pleural and vascular permeability, drawing fluid into the pleural space. Whereas cancerous cells that break away from solid tumors often die for lack of blood supply, it has been speculated that cancerous cells in the pleura may receive nourishment from the effusion fluid (Penz et al., 2017). Pleural metastasis can also interfere with lymph drainage, cause atelectasis, and reduce compliance of the chest wall and diaphragm, thus restricting oxygenation (Penz et al.).

The presence of MPE suggests a poor prognosis with estimates of median survival ranging from 3 to 24 months (Feller-Kopman et al., 2018). Using the Surveillance Epidemiology and End Results Registry, Shojaee et al. (2019) found the presence of MPE in 68,443 patients with small cell lung cancer to be an independent predictor of shorter survival.

Dyspnea and chest pain related to MPE can be severe and negatively impact quality of life (Psallidas et al., 2016), and dyspnea is typically, but not always progressive (Penz et al., 2017). The severity of dyspnea is affected by cardiac and pulmonary comorbidities, the size of the effusion, and the speed at which the effusion reaccumulates after it is drained (Ferreiro et al., 2020). Although rare, there is at least one published case that describes a man with small cell lung cancer who presented with a tension hydrothorax due to a rapidly reaccumulating MPE. Lung collapse, tracheal deviation, and distended neck veins were evident. The pleural fluid was drained one liter at a time over a 4-hour period, temporarily relieving symptoms; however, a 5.8-liter MPE accumulated in the following 48 hours (Porter et al., 2019).

## Tunneled/Indwelling Pleural Catheter

Insertion of a narrow, soft silicone IPC with a one-way valve has become a first-line therapy for MPE. These catheters allow palliative management of drainage at home with 89% to 100% improvement in symptoms (Wahla et al., 2019). Indwelling pleural catheters are minimally invasive, can be inserted in outpatient settings, and reduce the need for frequent travel to healthcare settings for thoracentesis (Feller-Kopman et al., 2018). Most importantly, a simple connection to a vacuum-drainage bottle allows the patient to maintain control over when drainage is needed and to effectively self-manage dyspnea and chest pain. Relief is usually immediate.

Infections related to IPCs are rare, but may include exit site infections and cellulitis, tunnel infections (that extend in from the exit site), purulent drainage, and empyema (infected, purulent drainage that collects in the chest around the catheter). Early identification of infection, cultures to identify the infecting organism(s), and antibiotics are important, especially given immunosuppressive effects of some therapies. It is typically necessary to remove the catheter for empyema (Chalhoub et al., 2018).

Fibrinous exudates and fibrous tissue may also build up and this may result in a reduction in drainage. In some cases, blockage of the IPC lumen and/or tip with fibrin may be reversed with injection of fibrinolytic agents such as a tissue plasminogen activator. Fibrous tissue can also build up in the pleural space causing the MPE fluid to separate into loculations and this may manifest as an increase in dyspnea and chest pain with a decrease or stoppage of drainage. Lung tissue may also become trapped and restricted by fibrinous tissue or tumor growth causing a decrease in drainage (Chalhoub et al., 2018). It is important to educate patients and caregivers to immediately report increases or decreases in the amount or characteristics of drainage.

Negative pressure with a vacuum bottle used to drain pleural effusions through an IPC can cause pain in some instances, especially with areas of trapped lung. Changes in severity of pain warrant immediate investigation (Chalhoub et al., 2018). Rapid removal of pleural fluid occasionally results in life-threatening reexpansion pulmonary edema evidenced by dyspnea, tachypnea, hypoxia, and cough (Meeker et al., 2016). No more than one liter should be drained at a time to prevent this complication (Carefusion, 2017; Porter et al., 2019). Instruct the patient or caregiver to clamp the drainage bottle and wait until symptoms resolve before resuming the drainage procedure. The physician should be notified of any concerns.

**Table.** ICD-10 Codes for Malignancy Diagnoses, Related Comorbidities, and PDGM Clinical Groupings

Diagnosis	ICD-10	HH ICD-10 Diagnosis Clinical Grouping & Comorbidity Adjustment
Malignant neoplasm of overlapping sites of right female breast	C50.811	MMTA_INFECT NEOPLASM (neoplasm 9)
Secondary metastasis of the right lung	C78.01	MMTA_INFECT NEOPLASM (neoplasm 17)
Secondary malignant neoplasm of pleura	C78.2	MMTA_INFECT NEOPLASM (neoplasm 17)
Secondary malignant neoplasm of bone	C79.51	MMTA_INFECT NEOPLASM (neoplasm 18)
Neoplasm (malignant) pleural effusion	J91.0	No
Neoplasm (cancer) related fatigue	R53.0	No
Anemia due to antineoplastic chemotherapy	D64.81	MMTA_INFECT NEOPLASM (circulatory 2)
Drug-induced polyneuropathy	G62.0	NEURO_REHAB (neurological 11)
Encounter for change or removal of drains	Z48.03	MMTA_AFTER
Encounter for palliative care	Z51.5	MMTA_OTHER

*Note*. Adapted from Centers for Medicare and Medicaid Services. (2019). *2020 ICD-10-CM Tabular List of Diseases and Injuries*. https://www.cms.gov/Medicare/Coding/ICD10/2020-ICD-10-CM

Centers for Medicare and Medicaid Services. (2021). Home Health PPS Grouper Software (HHGS). https://www.cms.gov/Medicare/Medicare-Fee-for-Service-Payment/HomeHealthPPS/CaseMixGrouperSoftware

The fluid of MPE is high in protein (up to 30 grams per liter) and loss of these proteins through drainage may contribute to malnutrition, cachexia, and third spacing of fluids. Symptom relief achieved from draining the MPE generally outweighs concern for protein loss via an IPC (Chalhoub et al., 2018), but these factors are important to consider when assessing nutritional needs with advanced cancer. In addition, it is also important to evaluate the patient's response to intravenous (IV) hydration fluids near end of life. If the patient with an MPE experiences increased chest pain and dyspnea during IV administration, the high protein content of the MPE may be third spacing water into the chest due to osmotic pressure gradients. This is the line that differentiates beneficial hydration from detrimental hydration. Although rare, subcutaneous metastasis along the catheter is possible. The probable mechanism is seeding due to cancer cell migration along the catheter tract. Seeding occurs in an estimated 5% of patients, but is most common with mesotheliomas (Chalhoub et al.).

## The Economics of Caring for Patients with an MPE: Home Care versus Hospital Care

The median cost of $42,376 for every 5.5-day hospital stay for patients with MPE reported by Taghizadeh et al. (2017) translates to $7,705 per day. The total bill in 2012 dollars for 126,825 patients with a primary or secondary diagnosis of MPE admitted to U.S. hospitals was over $5 billion dollars (Taghizadeh et al., 2017). We used the 2019 CMS PDGM Grouper that was previously available at https://www.cms.gov/Medicare/Medicare-Fee-for-Service-Payment/HomeHealthPPS/HH-PDGM, the ICD-10 diagnosis codes from Mrs. M's case history (Table), the Outcomes and Assessment Information Set responses consistent with her hospitalization risk, functional status, episode timing, and admission source to calculate case mix for Ms. M. We did not calculate wage index (this varies by area) or rural add on (which does not apply in this instance). The 30-day payment amount of $1,863.84 that Centers for Medicare and Medicaid Services (CMS) published in the calendar year 2021 Home Health Final Rule (CMS, 2020, p. 19) was used to estimate reimbursement.

Reimbursement for the first 30-day episode, with an early, institutional referral source and a clinical grouping of MMTA-Infectious Disease, Neoplasm (case mix 1.2948, HIPPS 2KB11, LUPA threshold 3), was estimated at $2,413.30. For the second 30 days of Mrs. M's home care, reimbursement was estimated at $1,455.10 (late, community, case mix 0.7807, HIPPS 3KB11, LUPA threshold 2).

It is clear, that caring for a patient with MPE at home saves a great deal of money. Whereas the cost for hospitalization was $7,705 per day, CMS will reimburse the home healthcare agency approximately $3,868, or $64.50 per day for a 60-day certification period. Mrs. M can remain in the comfort of her own home, cared for by people who love her. Quality of life is improved dramatically when the patient has the power to ease their suffering by draining the pleural effusion when experiencing shortness of breath and/or chest pain.

### Reimbursement for Pleural Vacuum Drainage Bottles Under PDGM

**Figure 2.**
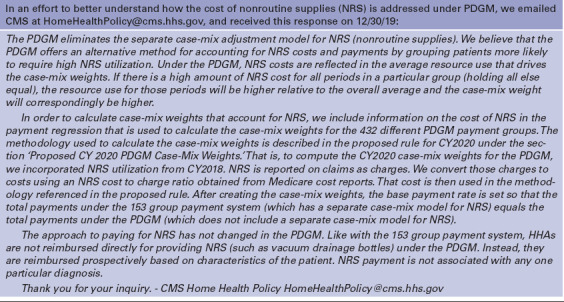
CMS explanation of reimbursement for nonroutine supplies under PDGM

For patients with Medicare, the pleural vacuum drainage bottles are considered a “bundled” nonroutine supply and the home healthcare agency is responsible for the costs of supplying this equipment. This is the case even if the patient is admitted for an unrelated issue and management of the IPC is not part of the home health plan of care. Payment for nonroutine supplies under PDGM is not based on supply costs or medical diagnosis; but is “reimbursed prospectively based on characteristics of the patient.” See Figure 2 for the CMS explanation received via email. Whereas the prior prospective payment system allowed for separate billing of $43.53 to $58.04 for each vacuum drainage bottle (CMS, 2019), the PDGM model does not.

### Estimating Costs

There are two pleural drainage systems in use in our geographical area, the PleurXTM brand (Benton, Dickinson and Company) and the Aspira® Drainage System. It is important to shop around. Our agency has recently worked with two of the top medical suppliers. Contract cost for supplier A is $107 to $125 per drainage kit (1, 1000 mL bottle) for the PleurX^TM^ and the cost with supplier B is $68 for the identical brand name kit. In a recent discussion sponsored by the National Association for Home Care and Hospice, participants reported costs of $500 to $1,023 for a case of 10 PleurX drainage bottles.

Using the $68 cost, if 18 vacuum bottles are used to drain three times a week for 21 days and daily for 9 days, vacuum bottles would cost $1,224 during the first 30-day episode. Using the calendar year 2020 skilled nursing cost per visit of $149.64 used by CMS (CMS, 2020, p. 21), the six planned visits for the first 30 days will be $897.84. With anticipated reimbursement calculated at $2,413.30 for 30-day episode 1, the balance is $291.46 (Figure 2).

As often occurs in patients with MPE, Mrs. M had an increase in pleural drainage over time and required more frequent drainage to achieve the same level of comfort. Anticipated reimbursement as calculated above is $1,455.10 for the second 30-day episode (and those that follow) as Mrs. M's status changed to late, community as she no longer meets the early, institutional referral source criteria. Case mix drops from 1.2948 to 0.7807.

The cost of daily drainage bottles is $2,040, leaving the agency with a loss of $585 for supplies alone. The nursing visits will cost $598.56 (for four) and increase to $1,047.48 if the three PRN visits are needed. Total losses for the second 30 days (and all subsequent 30-day episodes for recertifications) will range from $1,185 to $1,633 (Figure 2). Few agencies have the financial resources to sustain such losses.

## Getting Back to Mrs. M

After 21 days of home healthcare, Mrs. M was still feeling short of breath after draining 1,000 mL, so her oncologist sent a new order for daily drainage. The next day the nurse made a visit and notified the oncologist that she drained 850 mL before the drainage flow stopped. Pain management continued to be an issue and though it had improved with the addition of low-dose sustained release morphine, dexamethasone to reduce inflammation and pregabalin (Lyrica) to target neuropathic pain related to the spinal metastasis, Mrs. M voiced concerns about feeling overly sedated. The home care nurse discussed Mrs. M's concerns with the oncology team. Image guided stereotactic radiosurgery, delivery of small, highly focused beams of radiation (Zhang et al., 2020), was performed on the thoracic spine. The treatment was successful, and Mrs. M reported pain levels of 1 to 2 thereafter.

During the third 30-day episode (start of the first 60-day recertification), the amount of pleural drainage temporarily decreased with a corresponding increase in chest discomfort. This prompted a work-up to rule out IPC tip occlusion by fibrin, fibrous tissue build-up with loculations, and trapped lung. The IPC was found to be occluded and it was replaced by the interventional radiologist.

Toward the end of the third 30-day episode, Ms. M pain was minimal, and she had improved endurance and a desire to get stronger. A course of physical therapy was prescribed, and after a few weeks, Mrs. M reported a decrease in dyspnea and cancer-related fatigue as well as an improvement in strength and endurance. She and her family were independent with IPC management, there was no evidence of drug-related cardiotoxicity, pain was controlled, and she was independent with her home exercise program.

## A Time for Action—Advocating for Patients

Home healthcare patients or their caregivers are typically independent with care related to the IPC. As illustrated in this case study, the need for home care is due to complex needs associated with advanced malignancy and treatment. Under the Medicare prospective payment system, the agency could bill approximately $42 for each vacuum drainage bottle (CMS, 2019), but under PDGM this is no longer the case. Losses add up very quickly.

We are clearly not suggesting that any patient be denied care. However, bundling vacuum drainage bottles used for patients with MPEs (and malignant ascites) presents a serious financial burden for home healthcare agencies. Political action is needed to assist legislators to see the hardships caused by the loss of the partial reimbursement received for vacuum drainage bottles and other expensive nonroutine supplies bundled into the home healthcare payment. This is particularly problematic now, as agencies are experiencing unprecedented costs related to COVID-19 infection control and staffing.

Variations in contract costs from different suppliers is another issue that needs to be addressed collectively. How is it that supplier B charges 70% less than supplier A for an identical product? Why do some suppliers allow purchase of single-drainage kits, whereas others require purchase of a case of 10?

Could working through our home care and hospice organizations or other group efforts help to lower costs for everyone? How can we best communicate the special needs of home care patients to product developers? Is it possible, for example, to develop a lower cost, lightweight vacuum pleural device with a one-way valve that could be safely emptied several times a day? That would allow patients even greater control of their comfort as they could drain as often as needed as dyspnea worsens with even less risk of reexpansion pulmonary edema.

## Summary

This case illustrates the complex care and support needed by patients with MPE. These patients face numerous challenges while dealing with the consequences of advanced cancer, adverse effects related to treatment with antineoplasm agents, and dramatic social role changes for the family unit. Skilled assessments and patient/caregiver teaching facilitate rapid and effective interventions when complications do occur. Most importantly, patients with advanced cancer can more easily articulate their personal goals in a one patient/one nurse home setting so the things that are most important can be communicated across the care continuum.

Insertion of an IPC allows the patient to manage MPE related symptoms by allowing drainage of pleural effusions on an individualized timetable in the comfort of one's home. This intervention reduces human suffering for the patient as well as family members who would otherwise feel helpless watching their loved one struggling to breathe. Early identification and management of complications of advanced, metastatic cancer and MPE improve quality of life, and every patient deserves to have a qualified home healthcare team available to provide this service.

## INSTRUCTIONS Indwelling Pleural Catheters for Malignant Pleural Effusion: A Time for Action

### TEST INSTRUCTIONS

- Read the article. The test for this NCPD activity can be taken online at www.nursingcenter.com/ce/HHN. Tests can no longer be mailed or faxed.
- You will need to create a free login to your personal NCPD Planner account before taking online tests. Your planner will keep track of all your Lippincott Professional Development online NCPD activities for you.
- There is only one correct answer for each question. A passing score for this test is 7 correct answers. If you pass, you can print your certificate of earned contact hours and the answer key. If you fail, you have the option of taking the test again at no additional cost.
- For questions, contact Lippincott Professional Development: 1-800-787-8985.
- Registration Deadline: December 6, 2024.

### DISCLOSURE STATEMENT:

The authors and planners have disclosed no potential conflicts of interest, financial or otherwise.

### PROVIDER ACCREDITATION:

Lippincott Professional Development will award 2.0 contact hours for this nursing continuing professional development activity.

Lippincott Professional Development is accredited as a provider of nursing continuing professional development by the American Nurses Credentialing Center's Commission on Accreditation.

This activity is also provider approved by the California Board of Registered Nursing, Provider Number CEP 11749 for 2.0 contact hours. Lippincott Professional Development is also an approved provider of continuing nursing education by the District of Columbia, Georgia, and Florida CE, Broker #50-1223.

**Payment:** The registration fee for this test is $21.95.
